# Laparoscopic Toupet fundoplication: a alternative chance in elderly patients with GERD

**DOI:** 10.1186/1471-2482-13-S1-A21

**Published:** 2013-09-16

**Authors:** Antonio Ferronetti, Alfonso Canfora, Antonio Giugliano, Antonio Savanelli, Francesco Guida, Melania Battaglini Ciciriello, Bruno Amato, Giovanni Aprea

**Affiliations:** 1 Department of Gastroenterology, Endocrinology and Surgery, General Surgery Division, University “Federico II” of Naples, Via Pansini, 5 – 80131, Naples, Italy

## Background

Gastro-esophageal reflux disease (GERD) is one of the most frequent diseases of the upper gastro-enteric tract and incidence increases during lifetime [1]. In elderly patients [2] with a good LES basal tone and good peristalsis, evaluated with esophageal manometry, and even with a long exposure time fraction and a correlation between episodes of reflux and symptoms (SAP - Symptom Association Probability), evaluated with 24h pH-metry [3], severe and refractory symptoms or intolerance to a conservative treatment, had a good response after a laparoscopic 270° fundoplication (Toupet technique); Toupet's technique seems to offer good results such as Nissen-Rossetti fundoplication in terms of symptom control in a short-term evaluation [4-7].

## Materials and methods

We drove a prospective study in a period from March 2012 to March 2013, in which 13 elderly patients were treated with VLP Toupet fundoplication. Of these, 11 (84,6%) completed the follow up to six months, while 2 (16,4%) have not respected the post-operative protocol.

The inclusion criteria for the study were:

1. Patient age > 65

2. Long-term diagnosis of GERD

3. Severe symptoms with conservative treatment

4. Good LES basal tone and good esophageal motility

The patients have been preliminarily submitted to a 24 hours pH-metry and to an esophageal manometry.

The study foresaw a follow up of six months, that consisted in the repetition of 24 hours pH-metry and manometry one month and six months after the surgical intervention.

## Results and discussion

The results of study shows that after the intervention there are appreciable improvements, because all patients refer a meaningful decrease of symptoms severity, without post-operative disphagia. No manometric alterations and improvements of 24h ph-matric values were present in all cases.

Laparoscopic Toupet fundoplication seems to be a good alternative in selected elderly patients with severe symptoms of GERD, offering similar results of Nissen-Rossetti fundoplication [7].
